# Factors associated with HIV among young people attending Testing and Counseling Centers

**DOI:** 10.1590/0034-7167-2025-0089

**Published:** 2026-07-27

**Authors:** Pedro Augusto Bossonario, Rubia Laine de Paula Andrade-Gonçalves, Rafaele Oliveira Bonfim, Maria Amélia Zanon Ponce, Gabriela Tavares Magnabosco, Lucas Vinícius de Lima, Gabriel Zanin Sanguino, Aline Aparecida Monroe

**Affiliations:** I Universidade Estadual de Maringá. Maringá, Paraná, Brazil; II Universidade de São Paulo. Ribeirão Preto, São Paulo, Brazil; III Secretaria de Saúde de São José do Rio Preto. São José do Rio Preto, São Paulo, Brazil

**Keywords:** HIV, Adolescent, Young Adult, Sexual Behavior, Risk Factors., VIH, Adolescente, Adulto Joven, Conducta Sexual, Factores de Riesgo.

## Abstract

**Objectives:**

to analyze the factors associated with HIV among young people in a large Brazilian city.

**Methods:**

a case-control study was conducted with individuals aged 15 to 24 years old who sought services at the city’s Testing and Counseling Centers. Cases consisted of young people with a positive HIV test result, while controls were those with a negative result. Data were analyzed using descriptive statistics, chi-square test, and logistic regression.

**Results:**

men were 5.38 times more likely to have reactive HIV tests, sex workers were 31.5 times more likely to be infected with HIV, and those who sometimes or never used condoms were 2.43 times more likely to acquire the infection. Vaginal sex was a protective factor against HIV infection.

**Conclusions:**

factors associated with HIV should guide strategies that prioritize men, sex workers, and people with unprotected and non-vaginal sexual practices.

## RESUMEN

**Objetivos:**

analizar los factores asociados al VIH entre jóvenes de un municipio brasileño de porte grande.

**Métodos:**

es un estudio de caso-control, llevado a cabo con personas entre 15 y 24 años que buscaron Centros de Testeo y Consejería Municipal. Los casos estaban constituidos por jóvenes con VIH positivo, mientras que los controles por aquellos que presentaban resultado negativo. Los datos pasaron por análisis descriptivo, prueba Chi Cuadrado y regresión logística.

**Resultados:**

los hombres demostraron 5,38 veces más probabilidades de testeo positivo para el VIH; los profesionales del sexo, 31,5 veces más, y aquellos que a veces o nunca utilizaban preservativo, 2,43 veces más riesgo de contraer la infección. El sexo vaginal constituyó un factor de protección frente a la infección por el VIH.

**Conclusiones:**

es necesario crear estrategias sobre los factores asociados al VIH orientadas hacia hombres, profesionales del sexo y personas con prácticas sexuales desprotegidas y no vaginales.

## INTRODUCTION

According to estimates, 5.1 million adolescents and young adults were living with HIV worldwide in 2019, and despite a 46% decline in new infections in this population over the past decade, two out of every seven new HIV infections globally occur among individuals aged 15 to 24 years old^(1)^.

In Brazil, between 2022 and June 2023, 10,788 men and 2,443 women aged 15 to 24 years were diagnosed with new cases of HIV, the majority being between 20 and 24 years old^(2)^. In 2022, the detection rate of AIDS cases among young Brazilians aged 15 to 19 years old was 4.3 cases per 100,000 inhabitants, and among people aged 20 to 24 years, it was 21.2 cases per 100,000 inhabitants^(2)^. Between 2012 and 2022, 52,415 people aged 15 to 24 years old living with HIV progressed to AIDS, demonstrating the need to connect this population to health services, as well as to antiretroviral therapy^(2)^.

Young people are at the center of the HIV epidemic, as distinct factors increase the vulnerability of this population, such as low education, limited access to health services, early sexual initiation, coercion and sexual violence, as well as sexual exploitation^(3)^.

Therefore, laws and policies for young people’s access to HIV-related health services need to be rethought among countries, including this population in decision-making related to HIV in order to guarantee the expansion and sustainability of proposals outlined in the prevention of HIV infection, especially in countries with a higher prevalence of infection among young people^(1,4)^.

Furthermore, it is necessary to recognize the profile of this population, identifying those who are in greater conditions of vulnerability to acquire the HIV. In this regard, a systematic review showed that infection among young people in some studies was associated with female gender, older age, low educational level, Black individuals, multiple sexual partners, inconsistent condom use, alcohol consumption, and early sexual initiation^(5)^. However, none of the studies included in this review were conducted in Brazil, which motivated the present study, aiming to contribute to reducing the knowledge gaps on the topic.

## OBJECTIVES

To analyze the factors associated with HIV among young people in a large Brazilian city.

## METHODS

### Ethical aspects

This article was developed from a doctoral thesis. Its execution was approved by the Research Ethics Committee of the School of Nursing of Ribeirão Preto, University of São Paulo, after obtaining consent from the Municipal Health Department of Ribeirão Preto.

### Study desing, period and setting

This is an observational case-control study guided by the STROBE tool, whose recommendations were met.

The study was conducted in five Testing and Counseling Centers (TCCs) in a large city in São Paulo state, which perform serological testing for HIV, during the years 2018 to 2021. The TCCs are part of the Brazilian Unified Health System (SUS) and provide free care and follow-up for people living with sexually transmitted infections (STIs) and HIV^(6-8)^.

### Population or sample, inclusion and exclusion criteria

The study population consisted of young people who sought services at one of the five Testing and Counseling Centers (TCC). Among the cases, all young people who tested positive for HIV during the study period were included, while the controls included young people who tested negative for HIV. It should be noted that no variable was indicated for matching cases and controls. For the sample, 1 case was considered for every 4 controls, indicating a matching ratio of four. It is worth emphasizing that the controls were randomly selected and that, according to the Ministry of Health, the young population corresponds to those aged between 15 and 24 years^(9,10)^.

Data collection was carried out using the TCC Service Form, which is applied to individuals who attend the service for serological testing for HIV, syphilis, and hepatitis B and C and has 42 self-administered questions, distributed in eight sections: reason for seeking the service, identification (sociodemographic data), contact information, risk factors for hepatitis, tuberculosis (respiratory symptoms or contact), use of psychoactive drugs, STIs, and sexual behavior.

In addition, there are six sections of the instrument (population profile, type of exposure, testing, conduct, follow-up, and observations/closing) with 10 questions completed by health professionals, who use the responses of the individuals in the initial eight sections and the test results as a reference. The instrument contains dichotomous and multiple-choice answers.

These Service Forms were found in the archives of the five TCCs in the city and were transcribed entirely into a data collection instrument created using the Research Electronic Data Capture (REDCap platform).

### Analysis of results and statistics

The dependent variable in the study corresponded to the young people’s HIV infection status, while the independent variables were considered based on an understanding of sociodemographic and sexual behavior information, as well as the population segment, type of exposure, and testing. To ensure the comparability of the collected data, these were obtained from the same sources and simultaneously for positive cases and controls.

For data analysis, descriptive statistics techniques were first used with absolute and relative frequency distribution to describe the health history of the cases. Next, the association between the dependent and independent variables was analyzed using the Chi-square test. Variables with p-values ≥0.20 were included in the logistic regression model, keeping those with p-values <0.05 in the final model, with variable elimination occurring through the backward method. To verify the accuracy of the models, the obtained values were used to estimate the area under the ROC curve, where values from 0.90 to 0.99 are considered excellent; 0.80 to 0.89, good; 0.70 to 0.79, acceptable; and 0.51 to 0.69, poor^(11)^. All analyses were performed using the Statistica program from TIBCO Software Inc, version 14.0.0.15.

## RESULTS

Between 2018 and 2021, 420 young people were included in the study, with 57 seeking the service in 2018, 85 in 2019, 47 in 2020, and 231 in 2021. Of these, 84 young people tested positive for HIV.

Among the young participants in the study, 38.8% sought the testing center due to suspected STIs and 30.1% due to risky exposure; 37.7% had undergone their first HIV test, 39.6% their first syphilis test, and 41.0% their first hepatitis B and C test. Regarding the young people who tested positive for HIV, 32.9% also tested positive for syphilis and none tested positive for hepatitis B and C. Out of the young people who tested negative for HIV, 16.3% tested positive for syphilis and 1.6% tested positive for hepatitis B and C (Table 1).

**Table 1:** Distribution of young people served at Testing and Counseling Centers, according to health history, and serological testing for sexually transmitted infections and HIV, Ribeirão Preto, São Paulo, Brazil, 2018-2021

Variable		HIV reactive N = 84^*^	HIV non-reactive N = 336^*^	Total	*p* value
		n (%)	n (%)	n (%)	
**Reason for the search**	Suspected STI	30/84 (35.7)	130/328 (39.6)	160/412 (38.8)	0.511
	Risk exposure	28/84 (33.3)	96/328 (29.3)	124/412 (30.1)	0.469
**First time receiving care at a testing and counseling center (TCC) in my life**	Yes	22 (53.7)	99 (58.9)	121 (57.9)	0.540
	No	19 (46.3)	69 (41.1)	88 (42.1)	
**First HIV test in my life**	Yes	4 (25.0)	36 (40.0)	40 (37.7)	0.254
	No	12 (75.0)	54 (60.0)	66 (62.3)	
**First syphilis test in my life**	Yes	3 (23.1)	37 (42.1)	40 (39.6)	0.192
	No	10 (76.9)	51 (57.9)	61 (60.4)	
**First test for viral hepatitis** **in my life**	Yes	4 (33.3)	37 (42.1)	41 (41.0)	0.757^**^
	No	8 (66.7)	51 (57.9)	59 (59.0)	
**Syphilis**	Reactive	25 (32.9)	53 (16.3)	78 (19.4)	**<0.001**
	Non-reactive	51 (67.1)	273 (83.7)	324 (80.6)	
**Viral hepatitis**	Reactive	-	5 (1.6)	5 (1.3)	0.276
	Non-reactive	73 (100)	306 (98.4)	379 (98.7)	

* O N de jovens diferiu em todas as variáveis, uma vez que os dados em brancos/ignorados/não se aplicam não foram considerados nas análises;

** Teste Exato de Fisher; HIV - Vírus da imunodeficiência humana, IST - Infecções sexualmente transmissíveis; CTA - Centro de testagem e aconselhamento.

It should be noted that young people diagnosed with HIV had a statistically significant association with a positive syphilis test.

The average age of young people who tested positive for HIV was 21.6 (SD 2.2) years old and the median was 22 years, while among young people who tested negative for HIV, the average age was 21.0 (SD 2.3) years old and the median was 21 years.

Table 1 shows that most of the young people who went to the testing centers were men (66.4%), aged 20 to 24 years (78.1%), either white or mixed race (81.9%), single (88.4%), with complete high school education (62.3%), employed (62.3%), alcohol users (71.6%), and non-drug users (62.6%).

Applying the Chi-square or Fisher’s Exact Test, the following variables were identified as eligible for inclusion in the logistic regression model: gender, age group, employment, heterosexual population subgroup, MSM population subgroup, and sex worker population subgroup (Table 2).

**Table 2:** Distribution of young people according to sociodemographic profile and HIV test results, Ribeirão Preto, São Paulo, Brazil, 2018 to 2021

Variable		HIV reactive N = 84^*^	HIV non-reactive N = 336^*^	Total	*p* value
		n (%)	n (%)	n (%)	
**Gender identity**	Man	74 (88.1)	205 (61.0)	279 (66.4)	**<0.001**
	Woman	10 (11.9)	131 (39.0)	141 (33.6)	
**Age range**	15-19	13 (15.5)	79 (23.5)	92 (21.9)	0.111
	20-24	71 (84.5)	257 (76.5)	328 (78.1)	
**Race**	White	35 (46.1)	157 (48.9)	192 (48.4)	0.744^**^
	Black	12 (15.8)	56 (17.5)	68 (17.1)	
	Brown	29 (38.2)	104 (32.4)	133 (33.5)	
	Yellow/Indigenous	-	4 (1.3)	4 (1.0)	
**Matital Status**	Single	69 (93.2)	265 (87.2)	334 (88.4)	0.380^**^
	Married/Civil union	5 (6.8)	35 (11.5)	40 (10.6)	
	Separated/Widowed	-	4 (1.3)	4/378 (1.1)	
**Education**	None	6 (7.4)	26 (7.9)	32 (7.8)	0.971
	Complete Elementary School	17 (21.0)	70 (21.3)	87 (21.3)	
	High school diploma	52 (64.2)	203 (61.9)	255 (62.3)	
	Higher Eduaction	6 (7.4)	29 (8.8)	35 (8.6)	
**Work**	Yes	52 (81.2)	163 (58.0)	215 (62.3)	**<0.001**
	No	12 (18.8)	118 (42.0)	130 (37.7)	
**Alcohol use in the last 12 months**	Has never used it, or used it before but no longer uses it	23 (28.4)	93 (28.4)	116 (28.4)	0.994
	Uses	58 (71.6)	235 (71.6)	293 (71.6)	
**Use of illicit drugs in the last 12 months**	Has never used it, or used it before but no longer uses it	53 (65.4)	203 (61.9)	256 (62.6)	0.555
	Uses	28 (34.6)	125 (38.1)	153 (37.4)	

* The number of young people differed across all variables, as blank/missing/not applicable data were not included in the analyses;

** Fisher's Exact Test; HIV - Human immunodeficiency virus.

Regarding sexual behavior, it was possible to identify that most young people who went to the testing centers were heterosexual (53.6%) and that in the 12 months prior to data collection they had occasional partners (71.6%), 2 to 10 partners (62.5%), had relationships only with men (63.4%), had vaginal sex (61.9%), receptive oral sex (75.0%) and insertive oral sex (72.0%), and sometimes or never used condoms (72.7%). In this table, it is important to highlight that a large proportion of the cases that tested positive for HIV were men who have sex with men (79.5%) and practiced receptive (82.3%) and insertive (71.1%) anal sex in the 12 months prior to data collection.

By applying the Chi-square or Fisher’s Exact Test, the following variables were identified as eligible for inclusion in the logistic regression model: type of partnership, number of partners, sexual partnership, vaginal sex, receptive anal sex, insertive anal sex, insertive oral sex, and condom use (Table 3).

**Table 3:** Distribution of young people according to sexual behavior and HIV test results in the 12 months prior to data collection, Ribeirão Preto, São Paulo, Brazil, 2018 to 2021

Variable		HIV reactive N = 84^*^	HIV non-reactive N = 336^*^	Total	*p* value
		n (%)	n (%)	n (%)	
**Population segment**	Heterossexual	11/78 (14.1)	211/336 (62.8)	222/414 (53.6)	**<0.001**
	HSH	62/78 (79.5)	97/336 (28.9)	159/414 (38.4)	**<0.001**
	Transsexual	3/78 (3.9)	7/336 (2.1)	10/414 (2.4)	0.361^**^
	Sex worker	8/79 (10.1)	11/336 (3.3)	19/415 (4.6)	0.009^**^
**Type of partnership** **in the last 12 months**	Fixed	19 (13.2)	100 (29.8)	119 (28.5)	0.051
	Variable	22 (26.8)	117 (34.8)	139 (33.3)	
	Fixed and variable	41 (50.0)	119 (35.4)	160 (38.3)	
**Number of partnerships** **in the last 12 months**	1	15 (20.0)	77 (24.3)	92 (23.5)	0.013
	2	13 (13.3)	55 (17.4)	65 (16.6)	
	3 - 10	30 (40.0)	150 (47.3)	180 (45.9)	
	11 - 50	13 (17.3)	25 (7.9)	38 (9.7)	
	>50	7 (9.3)	10 (3.2)	17 (4.3)	
**Sexual partnership** **in the last 12 months**	Only men	69 (84.1)	196 (58.3)	265 (63.4)	**<0.001**
	Only women	5 (6.1)	108 (32.1)	113 (27.0)	
	Men and women	8 (9.8)	32 (9.5)	40 (9.6)	
**Sexual activity in** **the last 12 months**	Vaginal	16/79 (20.3)	241/336 (71.7)	257/415 (61.9)	**<0.001**
	Receptive anal	65/79 (82.3)	127/331 (38.4)	192/410 (46.8)	**<0.001**
	Insertive anal	54/76 (71.1)	115/330 (34.9)	169/406 (41.6)	**<0.001**
	Receptive oral	63/77 (81.8)	243/331 (73.4)	306/408 (75.0)	0.125
	Insertive oral	63/76 (82.9)	230/331 (69.5)	293/407 (72.0)	**0.019**
**Use of condoms** **in the last 12 months**	Always uses a condom	16 (20.5)	97 (28.9)	113 (27.3)	0.136
	Sometimes or never uses a condom	62 (79.5)	239 (71.1)	301 (72.7)	

* The number of young people differed across all variables, as blank/ignored/not applicable data were not considered in the analyses;

** Fisher's Exact Test; HIV - Human immunodeficiency virus.

In the logistic regression model (Table 4), among those who identified themselves as men, the odds of having a positive HIV test were 5.38 times higher than among women. It was also possible to identify that sex workers had 31.5 times more chance of having HIV infection, and those who sometimes or never used condoms had 2.43 times more chance of acquiring the infection. Vaginal sex was a protective factor against HIV infection.

**Table 4:** Logistic regression model of factors associated with HIV infection among young people seeking testing and counseling services, Ribeirão Preto, São Paulo, Brazil, 2018 to 2021

Variable	OR (CI 95%)	*p* value
Gender identity (men vs. women)	5.38 (1.05 - 27.53)	0.043
Sex worker (no vs. yes)	31.5 (5.12 - 193.25)	<0.001
Vaginal sex (no vs. yes)	0.09 (0.03 - 0.23)	<0.001
Condom use (sometimes or never vs. always)	2.43 (1.11 - 5.30)	0.026

*OR - odds ratio; 95% CI - 95% confidence interval.*

**Figure 1:**
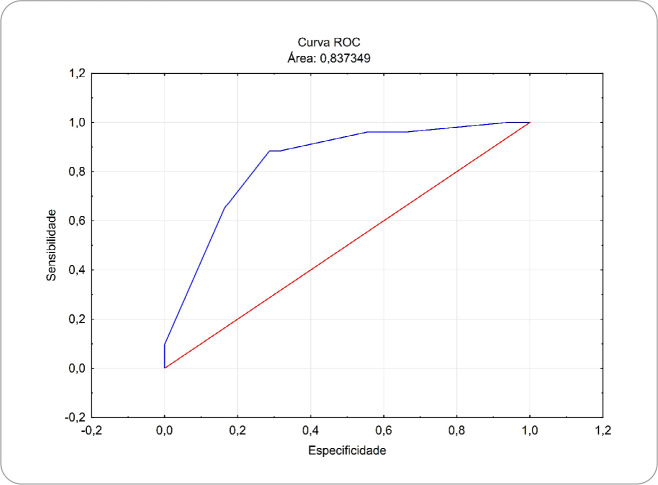
Area under the ROC curve in the modeling and validation datasets of factors associated with HIV infection in young people who sought testing and counseling centers, Ribeirão Preto, São Paulo, Brazil, 2018 to 2021

The area under the ROC curve was 0.837, which indicates that the model has a good capacity to predict HIV infection among young people (Figure 1).

## DISCUSSION

Analyzing the services provided by the STI testing centers during the investigated period, a significant decrease was observed during the first year of the Covid-19 pandemic in 2020, which may have occurred due to people’s fear of going to health units and contracting the disease, as well as the fact that several activities were interrupted due to the reorganization of actions and services to address the context experienced^(12)^. The decrease in services provided by health services can result in a worsening of chronic conditions, including HIV infection, generating an increase in the number of preventable deaths and causing a high economic and financial impact on health systems^(12)^.

Most cases that sought care at the STI testing centers had already undergone tests for HIV, syphilis, or viral hepatitis at some point in their lives. These data suggest both the accessibility of diagnostic actions for STIs in this population and the possibility of young people repeatedly exposing themselves to risky situations. This scenario is reinforced by the significant result of reactive tests for syphilis, converging with national data on a 2.6 times increase in the number of cases among adolescents aged 13 to 19 years between 2015 and 2022^(13,14)^.

Regarding factors associated with HIV, a higher prevalence was identified among men, aligning with the data found in the Brazilian literature, which shows a sex ratio of 28 men for every 10 women in 2020. This situation of vulnerability may be greater among men, since they comprise a large part of the key populations, such as gay men, men who have sex with men, people deprived of liberty, and drug users^(2,14)^.

Another risk factor for HIV found among young people was being a sex worker, a social group that should have guaranteed access to technological devices encompassed in Combined HIV Prevention, considering person-centered care, the local reality, and the singularities and preferences of each individual. Therefore, several actions must be developed to achieve the prevention of HIV transmission, which need to go beyond the distribution of condoms, including spaces and opportunities both within and outside institutions, as well as itinerant actions to reach young populations in different settings.

Inconsistent condom use was also identified as a factor associated with HIV infection among young people, demonstrating the relevance of this barrier method in reducing virus transmission. Since youth is a time when vulnerability to STIs may be increased with the initiation of sexual relations, in this sense, health education actions should be developed to build knowledge about STI prevention, as safe sex contributes to reducing HIV transmission, accompanied by the offer of serological testing^(15,16)^ and other technologies, such as lubricating gel and preand post-exposure prophylaxis.

Vaginal sexual intercourse was considered a protective factor against HIV among young people. This finding is consistent with the absence of women among the risk factors for HIV, and is also in line with the literature, which points to the practice of anal sex as being the most vulnerable to HIV infection^(17)^. In this sense, the importance of providing guidance to groups at higher risk of HIV transmission during consultations at HIV testing centers and other health services is highlighted, with emphasis on those who are part of Primary Health Care.

Understanding the factors associated with HIV among young people helps nurses develop health promotion and prevention actions against the virus, in addition to offering individual and collective care and counseling, enabling a reduction in the transmission of the virus and other STIs, while strengthening the construction of knowledge about timely diagnosis and treatment^(18)^.

Some variables were not associated with HIV among young people; however, reflections are necessary, since they can make the studied population more vulnerable. Among these variables, the age group of 20 to 24 years and alcohol use stand out, conditions that increase the vulnerability of this population, especially when associated with inconsistent condom use and multiple sexual partners^(19,20)^.

Understanding which young people are in a greater situation of vulnerability and exhibit risky behavior for HIV becomes important for adapting public policies that guarantee early diagnosis and timely treatment of HIV infection, in addition to directing the provision of health actions and services to the most vulnerable groups. For this, it is important that these initiatives are integrated and articulated with the continuum of care for people living with HIV, encompassing stages of diagnosis, linkage to services, retention in follow-up, adherence to treatment, and viral suppression^(21,22)^.

### Study limitations

The general limitations of this research include a possible information bias, due to the fact that the TCC (HIV testing and counseling center) intake form was not validated and may not be appropriate for the level of knowledge of the study participants. Furthermore, a possible selection bias is noted, since the controls were selected because they sought the same health services as the cases, services that are a reference for the diagnosis of STIs/HIV. It should also be noted that it was not possible to conduct a gender-based analysis to identify differences in sexual behavior between them.

### Contributions to the area

This study’s contribution facilitates the identification of populations most vulnerable to HIV infection in Testing and Counseling Centers, considering that these services represent entry points for the population, enabling both collective and individual care. This aspect is fundamental for timely diagnosis, early initiation of treatment, and monitoring of viral load, with a view to its suppression. Furthermore, the study allows for the construction of knowledge about the modes of HIV transmission, contributing to the adoption of effective strategies in reducing its spread.

## CONCLUSIONS

Based on the identification of factors associated with HIV among young people, strategies aimed at prevention, diagnosis, and timely treatment of the infection should be prioritized, especially among men, sex workers, and those who engage in unprotected and non-vaginal sexual practices. Such strategies should be aligned with Combined HIV Prevention and the Continuum of Care for people living with HIV.

## Data Availability

The research data are available within the article.

## References

[1] Conjunto das Nações Unidas (UNAIDS) (2021). UNAIDS data 2021.

[2] Ministério da Saúde (BR) (2023). Boletim Epidemiológico Sífilis.

[3] Dick B, Ferguson J, Ross DA., Ross DA, Dick B, Ferguson J, World Health Organization (2006). Preventing HIV/AIDS in young people: a systematic review of the evidence from developing countries.

[4] Dourado I, Silva LAV, Magno L, Brito AM, Jalil EM, Kendall C (2023). Combination HIV prevention for adolescent men who have sex with men and adolescent transgender women in Brazil: vulnerabilities, access to healthcare, and expansion of PrEP. Cad Saude Publica.

[5] Bossonario PA, Moreira RS, Costa AC, Lima VAS, Silva FN, Santos S (2022). Risk factors for HIV infection among adolescents and the youth: a systematic review. Rev Latino-Am Enfermagem.

[6] Moura JP, Ferreira ASAS. (2019). Soroprevalência em testagem itinerante para sífilis, HIV e hepatites.

[7] Beltrão RPL, Santos MMS, Lima AGT, Souza TS, Lima MC, Lopes CMB (2020). A assistência de saúde às pessoas vivendo com HIV/AIDS acompanhadas pelo Centro de Testagem e Aconselhamento. Res Soc Dev.

[8] Pereira SDS, Couto PLS, Rodrigues MMAS, Dos Santos NT, Pereira BDC, Flores TDS. (2020). Caracterização de usuários dos Centro de Testagem e Aconselhamento no Brasil: uma revisão integrativa. Rev Pró-UniverSUS.

[9] Ministério da Saúde (BR) (2010). Recomendações para terapia antirretroviral em adultos infectados pelo HIV - Suplemento III - Tratamento e Prevenção. Brasília, DF;.

[10] Instituto Brasileiro de Geografia e Estatística (IBGE) (2023). População no último censo: Ribeirão Preto.

[11] Carter JV, Pan J, Rai SN, Galandiuk S. (2016). ROC-ing along: evaluation and interpretation of receiver operating characteristic curves. Surgery.

[12] Mendes EV. (2020). Brasília.

[13] Godoy JA, Souza de Lima JA, Borges LL, Mesquita MM, Costa IR, Rocha HM (2021). Perfil epidemiológico da sífilis adquirida em pacientes de um laboratório clínico universitário em Goiânia-GO, no período de 2017 a 2019. Rev Bras Anal Clin.

[14] Ministério da Saúde (BR) (2023). Boletim Epidemiológico HIV.

[15] Ferro LD, Martins LL, Andrade Ferreira E, Leite PM, Oliveira Machado PHR, Assis LDMG (2021). Prevalência de coinfecção por sífilis e HIV em adolescentes no Brasil. Braz J Health Rev.

[16] Sousa LRM, Pinheiro TF, Reis RK, Carvalho WMES, Gir E, Shimizu HE. (2023). Inconsistent use of male condoms among HIV-negative men who have sex with other men. Rev Latino-Am Enfermagem.

[17] Centers for Disease Control and Prevention (CDC) (2015). National Center for HIV/AIDS, Viral Hepatitis, STD, and TB Prevention. Division of HIV/AIDS Prevention. HIV Risk Behaviors.

[18] Augusto PS, Silva RL, Costa LHP, Gomes LFP, Souza RS, Oliveira TCA (2024). Health management of an HIV testing and counseling center: nursing contributions. Rev Bras Enferm.

[19] Mesquita AL, Melo ES, Costa CRB, Pontes PS, Gir E, Reis RK. (2020). Consumo de álcool em pessoas vivendo com HIV e suas implicações para os desfechos clínicos. Rev Eletr Enferm.

[20] Garcia EC, Costa IR, Oliveira RCD, Silva CRL, Góis ARS, Abrão FMS. (2021). Social representations of adolescents about HIV/AIDS transmission in sexual relations: vulnerabilities and risks. Esc Anna Nery.

[21] Ministério da Saúde (BR) (2024). Protocolo clínico e diretrizes terapêuticas para manejo da infecção pelo HIV em crianças e adolescentes: módulo 1: diagnóstico, manejo e acompanhamento de crianças expostas ao HIV.

[22] Ministério da Saúde (BR) (2024). Protocolo clínico e diretrizes terapêuticas para manejo da infecção pelo HIV em crianças e adolescentes: módulo 2: diagnóstico, manejo e tratamento de crianças e adolescentes vivendo com HIV.

