# Supplementary material for: Factors associated with HIV among young people attending Testing and Counseling Centers
Source: Rev Bras Enferm. 2026 Jul 27;79:e20250089. doi: 10.1590/0034-7167-2025-0089 (PMC13411319; doi:10.1590/0034-7167-2025-0089)
Supplement: Translation

# Translation: Fatores associados ao HIV entre jovens atendidos em Centros de Testagem e Aconselhamento

**DOI:** 10.1590/0034-7167-2025-0089pt

**Authors:** Pedro Augusto Bossonario, Rubia Laine de Paula Andrade-Gonçalves, Rafaele Oliveira Bonfim, Maria Amélia Zanon Ponce, Gabriela Tavares Magnabosco, Lucas Vinícius de Lima, Gabriel Zanin Sanguino, Aline Aparecida Monroe

**Affiliations:** I Universidade Estadual de Maringá. Maringá, Paraná, Brasil; II Universidade de São Paulo. Ribeirão Preto, São Paulo, Brasil; III Secretaria de Saúde de São José do Rio Preto. São José do Rio Preto, São Paulo, Brasil

**Keywords:** HIV, Adolescente, Adulto Jovem, Comportamento Sexual, Fatores de Risco.

## Abstract

**Objetivos:**

analisar os fatores associados ao HIV entre jovens de um município brasileiro de grande porte.

**Métodos:**

estudo de caso-controle, realizado com pessoas de 15 a 24 anos que procuraram os Centros de Testagem e Aconselhamento do município. Os casos foram constituídos por jovens com resultado positivo ao teste de HIV, enquanto os controles constituíram-se por aqueles que apresentaram resultado negativo. Os dados passaram por análise descritiva, teste qui-quadrado e regressão logística.

**Resultados:**

homens tiveram 5,38 vezes mais chances de testes reagentes para HIV, profissionais do sexo apresentaram 31,5 vezes mais chances de infecção por HIV e aqueles que às vezes ou nunca utilizavam preservativo possuíam 2,43 vezes mais chance de adquirir a infecção. O sexo vaginal constituiu fator de proteção para infecção por HIV.

**Conclusões:**

fatores associados ao HIV devem orientar estratégias que priorizem homens, profissionais do sexo e pessoas com práticas sexuais desprotegidas e não vaginais.

## INTRODUÇÃO

Segundo estimativas, 5,1 milhões de adolescentes e adultos jovens viviam com HIV no mundo em 2019 e, apesar do declínio de 46% nas novas infecções na última década, a cada sete novas infecções pelo vírus no mundo, duas ocorrem entre indivíduos de 15 a 24 anos^(1)^.

No Brasil, entre 2022 até junho de 2023, 10.788 homens e 2.443 mulheres com idade entre 15 e 24 anos tiveram diagnóstico de casos novos de HIV, sendo a maioria com idade entre 20 e 24 anos^(2)^. Em 2022, a taxa de detecção de casos de aids entre jovens brasileiros de 15 a 19 anos foi de 4,3 casos a cada 100 mil habitantes e entre as pessoas com 20 a 24 anos foi de 21,2 casos a cada 100 mil habitantes^(2)^. No período de 2012 a 2022, 52.415 pessoas entre 15 e 24 anos que viviam com HIV evoluíram para aids, o que demonstra a necessidade de vinculação dessa população aos serviços de saúde, bem como à terapia antirretroviral^(2)^.

As pessoas jovens estão no centro da epidemia do HIV, uma vez que distintos fatores aumentam a vulnerabilidade dessa população, como: baixa educação, baixo acesso aos serviços de saúde, início sexual precoce, coerção e violência sexual, bem como exploração sexual^(3)^.

Dessa forma, as leis e políticas para acesso de jovens aos serviços de saúde direcionados ao HIV precisam ser repensadas entre os países, inserindo tal população em tomadas de decisões relacionadas ao HIV a fim de garantir a ampliação e a sustentabilidade de propostas elencadas na prevenção da infecção pelo vírus, principalmente em países com maior prevalência da infecção entre jovens^(1,4)^.

Além disso, é necessário o reconhecimento do perfil dessa população, identificando aqueles que estão em maiores condições de vulnerabilidade ao HIV. Nesse sentido, uma revisão sistemática mostrou que a infecção entre jovens, em alguns estudos, esteve associada ao sexo feminino, à maior idade dos jovens, baixa escolaridade, pessoas negras, múltiplas parcerias sexuais, uso inconsistente de preservativos, consumo de álcool e início sexual precoce^(5)^. No entanto, nenhum dos estudos incluídos na referida revisão foi conduzido no Brasil, o que motivou a realização do presente estudo, visando contribuir para a redução das lacunas de conhecimento sobre o tema.

## OBJETIVOS

Analisar os fatores associados ao HIV entre jovens de um município brasileiro de grande porte.

## MÉTODOS

### Aspectos éticos

O presente artigo foi elaborado a partir da tese de doutorado, cuja execução foi assentida pelo Comitê de Ética em Pesquisa da Escola de Enfermagem de Ribeirão Preto, da Universidade de São Paulo após consentimento da Secretaria Municipal de Saúde de Ribeirão Preto.

### Desenho, período e local do estudo

Trata-se de um estudo observacional do tipo caso-controle norteado pela ferramenta STROBE, a qual teve as recomendações atendidas.

O estudo foi realizado em cinco Centros de Testagem e Aconselhamento (CTA) de um município paulista de grande porte, os quais realizam testagem sorológica para HIV, durante os anos de 2018 a 2021. Os CTA estão inseridos no Sistema Único de Saúde (SUS) brasileiro e possibilitam o acolhimento e seguimento das pessoas que vivem com infecção sexualmente transmissível (IST) e HIV de forma gratuita^(6-8)^.

### População ou amostra, critérios de inclusão e exclusão

A população do estudo contou com jovens que procuraram um dos cinco Centros de Testagem e Aconselhamento (CTA). Entre os casos, foram considerados todos os jovens que apresentaram resultado positivo ao teste de HIV no período estudado, enquanto os controles incluíram jovens que apresentaram resultado negativo ao teste de HIV. Destaca-se que não foi indicada nenhuma variável para pareamento dos casos e controles. Para amostra, foram considerados 1 caso para 4 controles, o que indica razão de pareamento igual a quatro. Vale ressaltar que os controles foram selecionados aleatoriamente e que, de acordo com o Ministério da Saúde, a população jovem corresponde àquela com idade entre 15 e 24 anos^(9,10)^.

A coleta de dados foi realizada por meio da Ficha de Atendimento do CTA, a qual é aplicada aos sujeitos que comparecem ao serviço para testagem sorológico de HIV, sífilis e hepatites B e C e possui 42 questões autoaplicáveis, distribuídas em oito seções: busca pelo serviço, identificação (dados sociodemográficos), informações para contato, fatores de risco para hepatites, tuberculose (sintomas respiratórios ou contato), uso de drogas psicoativas, IST e comportamento sexual.

Ainda, há seis seções do instrumento (recorte populacional, tipo de exposição, testagem, conduta, retorno e observações/encerramento) com 10 questões preenchidas pelos profissionais de saúde, os quais tomam como referência a resposta das pessoas nas oito seções iniciais e os resultados dos exames. O instrumento contém respostas dicotômicas e de múltiplas escolhas.

Tais Fichas de Atendimento encontravam-se nos arquivos dos cinco CTA do município e foram transcritas integralmente a um instrumento elaborado para coleta de dados que foi criado por meio da plataforma intitulada *Research Electronic Data Capture* (REDCap).

### Análise dos resultados e estatísticas

A variável dependente do estudo correspondeu ao status dos jovens em relação à infecção pelo HIV, enquanto as variáveis independentes foram consideradas a partir da compreensão das informações sociodemográficas e de comportamento sexual, bem como do recorte populacional, tipo de exposição e testagem. Para assegurar a comparabilidade dos dados coletados, estes foram obtidos das mesmas fontes e de maneira simultânea para os casos e controles.

Para análise dos dados, primeiramente, utilizou-se técnicas de estatística descritiva com distribuição de frequência absoluta e relativa para descrever o histórico de saúde dos casos. Em seguida, analisou-se a associação da variável dependente e independentes por meio do teste Qui-quadrado. Variáveis com resultados de valor de p≥0,20 foram incluídas no modelo de regressão logística, mantendo, no modelo final, aquelas com valor de p<0,05, sendo que a eliminação das variáveis ocorreu pelo método de *Backward*. Para verificar a acurácia dos modelos, os valores obtidos foram usados para estimar a área sob a curva ROC, sendo que valores de 0,90 a 0,99 são considerados excelentes; de 0,80 a 0,89, bons; de 0,70 a 0,79, aceitáveis; e de 0,51 a 0,69, ruins^(11)^. Todas as análises foram realizadas por meio do programa Statistica da TIBCO Software Inc, versão 14.0.0.15.

## RESULTADOS

No período de 2018 a 2021, 420 jovens foram incluídos no estudo, sendo que 57 buscaram o serviço em 2018, 85 em 2019, 47 em 2020 e 231 em 2021. Dentre estes, 84 jovens apresentaram resultado reagente para HIV.

Dentre os jovens participantes do estudo, 38,8% procuraram o CTA por suspeita de IST e 30,1% por exposição de risco, 37,7% haviam realizado o primeiro teste de HIV na vida, 39,6%, o primeiro teste de sífilis e 41,0%, o primeiro teste de hepatite B e C. Quanto aos jovens com teste reagente para HIV, 32,9% também foram reagentes para sífilis e nenhum reagente para hepatite B e C. Dos jovens com teste não reagente para HIV, 16,3% foram reagentes para sífilis e 1,6% foi reagente para hepatite B e C (Tabela 1).

**Tabela 1:** Distribuição de jovens atendidos nos Centros de Testagem e Aconselhamento, segundo histórico de saúde, e testagem sorológica para infecções sexualmente transmissíveis e HIV, Ribeirão Preto, São Paulo, Brasil, 2018-2021

Variável		HIV reagente N = 84^*^	HIV não reagente N = 336^*^	Total	Valor de *p*
		n (%)	n (%)	n (%)	
**Motivo da procura**	Suspeita IST	30/84 (35,7)	130/328 (39,6)	160/412 (38,8)	0,511
	Exposição de risco	28/84 (33,3)	96/328 (29,3)	124/412 (30,1)	0,469
**1º atendimento em CTA na vida**	Sim	22 (53,7)	99 (58,9)	121 (57,9)	0,540
	Não	19 (46,3)	69 (41,1)	88 (42,1)	
**Primeiro teste na vida de HIV**	Sim	4 (25,0)	36 (40,0)	40 (37,7)	0,254
	Não	12 (75,0)	54 (60,0)	66 (62,3)	
**Primeiro teste na vida de sífilis**	Sim	3 (23,1)	37 (42,1)	40 (39,6)	0,192
	Não	10 (76,9)	51 (57,9)	61 (60,4)	
**Primeiro teste na vida de hepatites virais**	Sim	4 (33,3)	37 (42,1)	41 (41,0)	0,757^**^
	Não	8 (66,7)	51 (57,9)	59 (59,0)	
**Sífilis**	Reagente	25 (32,9)	53 (16,3)	78 (19,4)	**<0,001**
	Não reagente	51 (67,1)	273 (83,7)	324 (80,6)	
**Hepatites virais**	Reagente	-	5 (1,6)	5 (1,3)	0,276
	Não reagente	73 (100)	306 (98,4)	379 (98,7)	

* O N de jovens diferiu em todas as variáveis, uma vez que os dados em brancos/ignorados/não se aplicam não foram considerados nas análises;

** Teste Exato de Fisher; HIV - Vírus da imunodeficiência humana, IST - Infecções sexualmente transmissíveis; CTA - Centro de testagem e aconselhamento.

Cabe destacar que os jovens diagnosticados com HIV tiveram associação estatisticamente significativa com teste reagente para sífilis.

A idade média dos jovens que tiveram teste reagente para HIV foi de 21,6 (dp 2,2) anos e mediana de 22 anos, enquanto entre os jovens não reagentes para HIV foi de 21,0 (dp 2,3) anos e mediana de 21 anos.

Pela tabela 1, é possível identificar que a maioria dos jovens atendidos nos CTA eram homens (66,4%), com idade de 20 a 24 anos (78,1%), cor da pele branca ou parda (81,9%), solteiros (88,4%), com ensino médio completo (62,3%), trabalhavam (62,3%), faziam uso de álcool (71,6%) e não faziam uso de drogas (62,6%).

Ao aplicar o Qui-quadrado ou Teste Exato de Fisher, identificou-se as seguintes variáveis elegíveis para a inclusão no modelo de regressão logística: gênero, faixa etária, trabalho, recorte populacional heterossexual, recorte populacional HSH, recorte populacional profissional do sexo (Tabela 2).

**Tabela 2:** Distribuição de jovens segundo perfil sociodemográfico e resultado do teste para HIV, Ribeirão Preto, São Paulo, Brasil, 2018 a 2021

Variável		HIV reagente N = 84^*^	HIV não reagente N = 336^*^	Total	Valor de *p*
		n (%)	n (%)	n (%)	
**Identidade de gênero**	Homem	74 (88,1)	205 (61,0)	279 (66,4)	**<0,001**
	Mulher	10 (11,9)	131 (39,0)	141 (33,6)	
**Faixa etária**	15-19	13 (15,5)	79 (23,5)	92 (21,9)	0,111
	20-24	71 (84,5)	257 (76,5)	328 (78,1)	
**Raça**	Branca	35 (46,1)	157 (48,9)	192 (48,4)	0,744^**^
	Preta	12 (15,8)	56 (17,5)	68 (17,1)	
	Parda	29 (38,2)	104 (32,4)	133 (33,5)	
	Amarela/Indígena	-	4 (1,3)	4 (1,0)	
**Situação conjugal**	Solteiro	69 (93,2)	265 (87,2)	334 (88,4)	0,380^**^
	Casado/União estável	5 (6,8)	35 (11,5)	40 (10,6)	
	Separado/Viúvo	-	4 (1,3)	4/378 (1,1)	
**Escolaridade**	Nenhuma	6 (7,4)	26 (7,9)	32 (7,8)	0,971
	Fundamental completo	17 (21,0)	70 (21,3)	87 (21,3)	
	Médio completo	52 (64,2)	203 (61,9)	255 (62,3)	
	Superior completo	6 (7,4)	29 (8,8)	35 (8,6)	
**Trabalho**	Sim	52 (81,2)	163 (58,0)	215 (62,3)	**<0,001**
	Não	12 (18,8)	118 (42,0)	130 (37,7)	
**Álcool nos últimos 12 meses**	Nunca usou ou já usou e não usa mais	23 (28,4)	93 (28,4)	116 (28,4)	0,994
	Usa	58 (71,6)	235 (71,6)	293 (71,6)	
**Uso de drogas ilícitas nos últimos 12 meses**	Nunca usou ou já usou e não usa mais	53 (65,4)	203 (61,9)	256 (62,6)	0,555
	Usa	28 (34,6)	125 (38,1)	153 (37,4)	

* O N de jovens diferiu em todas as variáveis, uma vez que os dados em brancos/ignorados/não se aplicam não foram considerados nas análises;

** Teste Exato de Fisher; HIV - Vírus da imunodeficiência humana.

Quanto ao comportamento sexual, foi possível identificar que a maioria dos jovens atendidos nos CTA eram heterossexuais (53,6%) e que nos 12 meses anteriores à coleta de dados tiveram parceria eventual (71,6%), de 2 a 10 parcerias (62,5%), se relacionam somente com homens (63,4%), tiveram relação sexual vaginal (61,9%), oral receptivo (75,0%) e oral insertivo (72,0%) e às vezes ou nunca fizeram uso de preservativo (72,7%). Nesta tabela, é importante ressaltar que grande parcela dos casos que foram reagentes ao HIV eram homens que faziam sexo com homens (79,5%) e praticaram o sexo anal receptivo (82,3%) e insertivo (71,1%) nos 12 meses anteriores à coleta dos dados.

Ao aplicar o Qui-quadrado ou Teste Exato de Fisher, identificou-se as seguintes variáveis elegíveis para a inclusão no modelo de regressão logística: tipo de parceria, número de parceria, parceria sexual, sexo vaginal, sexo anal receptivo, sexo anal insertivo, sexo oral insertivo e uso de preservativo (Tabela 3).

**Tabela 3:** Distribuição de jovens segundo o comportamento sexual e resultado do teste para HIV nos 12 meses anteriores à coleta de dados, Ribeirão Preto, São Paulo, Brasil, 2018 a 2021

Variável		HIV reagente N = 84^*^	HIV não reagente N = 336^*^	Total	Valor de *p*
		n (%)	n (%)	n (%)	
**Recorte populacional**	Heterossexual	11/78 (14,1)	211/336 (62,8)	222/414 (53,6)	**<0,001**
	HSH	62/78 (79,5)	97/336 (28,9)	159/414 (38,4)	**<0,001**
	Transexual	3/78 (3,9)	7/336 (2,1)	10/414 (2,4)	0,361^**^
	Profissional do sexo	8/79 (10,1)	11/336 (3,3)	19/415 (4,6)	0,009^**^
**Tipo de parceria nos** **últimos 12 meses**	Fixa	19 (13,2)	100 (29,8)	119 (28,5)	0,051
	Eventual	22 (26,8)	117 (34,8)	139 (33,3)	
	Fixa e eventual	41 (50,0)	119 (35,4)	160 (38,3)	
**Número de parcerias nos últimos 12 meses**	1	15 (20,0)	77 (24,3)	92 (23,5)	0,013
	2	13 (13,3)	55 (17,4)	65 (16,6)	
	3 - 10	30 (40,0)	150 (47,3)	180 (45,9)	
	11 - 50	13 (17,3)	25 (7,9)	38 (9,7)	
	>50	7 (9,3)	10 (3,2)	17 (4,3)	
**Parceria sexual nos** **últimos 12 meses**	Somente homens	69 (84,1)	196 (58,3)	265 (63,4)	**<0,001**
	Somente mulheres	5 (6,1)	108 (32,1)	113 (27,0)	
	Homens e mulheres	8 (9,8)	32 (9,5)	40 (9,6)	
**Prática sexual nos** **últimos 12 meses**	Vaginal	16/79 (20,3)	241/336 (71,7)	257/415 (61,9)	**<0,001**
	Anal receptivo	65/79 (82,3)	127/331 (38,4)	192/410 (46,8)	**<0,001**
	Anal insertivo	54/76 (71,1)	115/330 (34,9)	169/406 (41,6)	**<0,001**
	Oral receptivo	63/77 (81,8)	243/331 (73,4)	306/408 (75,0)	0,125
	Oral insertivo	63/76 (82,9)	230/331 (69,5)	293/407 (72,0)	**0,019**
**Uso de preservativo** **nos último 12 meses**	Sempre utiliza preservativo	16 (20,5)	97 (28,9)	113 (27,3)	0,136
	Às vezes ou nunca utiliza preservativo	62 (79,5)	239 (71,1)	301 (72,7)	

* O N de jovens diferiu em todas as variáveis, uma vez que os dados em brancos/ignorados/não se aplicam não foram considerados nas análises;

** Teste Exato de Fisher; HIV - Vírus da imunodeficiência humana.

No modelo de regressão logística (Tabela 4), entre aqueles que se declaram homens, as chances de ter teste reagente para HIV foi 5,38 vezes maior do que entre as mulheres. Foi possível identificar, ainda, que profissionais do sexo tinham 31,5 vezes mais chance de ter infecção por HIV e aqueles que às vezes ou nunca utilizavam preservativo possuíam 2,43 vezes mais chance de adquirir a infecção. O sexo vaginal constituiu fator de proteção para infecção de HIV.

**Tabela 4:** Modelo de regressão logística dos fatores associados à infecção pelo HIV entre jovens que buscaram os Centros de Testagem e Aconselhamento, Ribeirão Preto, São Paulo, Brasil, 2018 a 2021

Variável	OR (IC 95%)	Valor de *p*
Identidade de gênero (homens vs. mulher)	5,38 (1,05 - 27,53)	0,043
Profissional do sexo (não vs. sim)	31,5 (5,12 - 193,25)	<0,001
Sexo vaginal (não vs. sim)	0,09 (0,03 - 0,23)	<0,001
Uso de preservativo (às vezes ou nunca vs. sempre)	2,43 (1,11 - 5,30)	0,026

*OR - odds ratio; IC95% - intervalo de confiança 95%.*

A área sob a curva ROC foi de 0,837, o que denota que o modelo possui uma capacidade boa de predizer a infecção pelo HIV entre jovens (Figura 1).

**Figura 1:** Área sob a curva ROC nos conjuntos de dados de modelagem e validação dos fatores associados à infecção pelo HIV em jovens que buscaram os Centros de Testagem e Aconselhamento, Ribeirão Preto, São Paulo, Brasil, 2018 a 2021

## DISCUSSÃO

Ao analisar os atendimentos realizados pelos serviços de CTA durante o período investigado, observou-se importante queda durante o primeiro ano da pandemia da Covid-19 em 2020, o que pode ter ocorrido devido ao medo das pessoas em se deslocarem até as unidades de saúde e contraírem a doença, bem como pelo fato de que diversas atividades foram interrompidas em função da reorganização das ações e serviços para o enfrentamento do contexto vivenciado^(12)^. A diminuição nos atendimentos realizados pelos serviços de saúde pode resultar em um agravamento das condições crônicas e, dentre elas, da infecção pelo HIV, gerando aumento no número de casos de óbitos evitáveis e causando alto impacto econômico-financeiro nos sistemas de saúde^(12)^.

A maioria dos casos que procuraram atendimento entre os CTA já havia realizado, em algum momento da vida, testes para HIV, sífilis ou hepatites virais. Esses dados sugerem tanto a acessibilidade às ações de diagnóstico às IST nessa população quanto a possibilidade de jovens estarem, repetidamente, expondo-se às situações de risco. Esse cenário é reforçado pelo expressivo resultado de testes reagentes para sífilis, convergindo com os dados nacionais acerca do aumento de 2,6 vezes no número de casos entre adolescentes de 13 a 19 anos no período de 2015 a 2022^(13,14)^.

Quanto aos fatores associados ao HIV, identificou-se maior prevalência entre os homens, alinhando-se aos dados encontrados na literatura brasileira, a qual apresenta a razão entre os sexos de 28 homens a cada 10 mulheres no ano de 2020. Essa situação de vulnerabilidade pode estar maior entre os homens, uma vez que eles compõem grande parte do contingente das populações-chave, como gays, homens que fazem sexo com homens, pessoas privadas de liberdade e usuários de drogas^(2,14)^.

Outro fator de risco ao HIV encontrado entre os jovens foi ser profissional do sexo, grupo social que deve ter garantida a oferta de dispositivos tecnológicos abarcados na Prevenção Combinada ao HIV, considerando o cuidado centrado na pessoa, a realidade local e as singularidades e preferências de cada indivíduo. Assim, diversas ações devem ser desenvolvidas a fim de alcançar a prevenção da transmissão ao HIV, as quais necessitam extrapolar a distribuição de preservativo, incluindo espaços e oportunidades intra e extramuros, bem como ações itinerantes para o alcance de populações jovens em diferentes cenários.

A utilização inconsistente de preservativos foi identificada também como um fator associado à infecção pelo HIV entre jovens, demonstrando a relevância deste método de barreira na diminuição da transmissão do vírus. Uma vez que a juventude apresenta-se como um momento em que a vulnerabilidade às IST pode estar aumentada com a iniciação das relações sexuais, é necessário que ações de educação em saúde sejam desenvolvidas para a construção do conhecimento acerca da prevenção das IST, pois o sexo seguro contribui para a redução da transmissão do HIV, acompanhadas da oferta de testagem sorológicos^(15,16)^ e outras tecnologias, como gel lubrificante e profilaxias pré e pós exposição.

As relações sexuais praticadas por via vaginal foram consideradas como fator de proteção ao HIV entre os jovens. Esse achado está em consonância com a ausência de mulheres entre os fatores de risco ao HIV, além de estar em convergência com a literatura, que aponta a prática do sexo anal sendo a de maior vulnerabilidade à infecção pelo HIV^(17)^. Nesse sentido, destaca-se a importância da realização de orientações a grupos com maiores riscos de transmissão do HIV durante as consultas em CTA e outros serviços de saúde, com ênfase naqueles que fazem parte da Atenção Primária à Saúde.

A compreensão dos fatores associados ao HIV entre jovens colabora para que a enfermagem desenvolva ações de promoção da saúde e prevenção ao vírus, além de oferecer atendimentos e aconselhamentos individuais e coletivos, possibilitando a diminuição da transmissão do vírus e outras IST, ao mesmo tempo em que fortalece a construção do conhecimento acerca do diagnóstico oportuno e tratamento^(18)^.

Houve variáveis que não tiveram associação com o HIV entre os jovens, entretanto, reflexões são necessárias, uma vez que podem vulnerabilizar a população estudada. Dentre essas variáveis, destacam-se a faixa etária de 20 a 24 anos e o uso de álcool, condições que aumentam a vulnerabilidade dessa população, principalmente quando estão associadas ao uso de preservativos de forma inconsistente e a múltiplas parcerias sexuais^(19,20)^.

Compreender quais jovens estão em maior situação de vulnerabilidade e apresentam comportamento de risco para o HIV torna-se importante para a adequação de políticas públicas que garantam o diagnóstico precoce e o tratamento oportuno da infecção pelo HIV, além de direcionar a oferta de ações e serviços de saúde para os grupos mais vulneráveis. Para isso, é importante que tais iniciativas estejam integradas e articuladas à linha do cuidado contínuo às pessoas que vivem com HIV, abrangendo etapas de diagnóstico, vinculação ao serviço, retenção no seguimento, adesão ao tratamento e supressão viral^(21,22)^.

### Limitações do estudo

Como limitações gerais da presente pesquisa, destaca-se um possível viés de informação, em função da ficha de atendimento do CTA não ter sido validada e pela possibilidade de não estar adequada ao nível de conhecimento dos participantes do estudo. Ainda, pontua-se um possível viés de seleção, uma vez que os controles foram selecionados por buscarem os mesmos serviços de saúde que os casos, serviços estes que são referência para o diagnóstico de IST/HIV. Cabe destacar que não foi possível a realização de uma análise por gênero para identificar diferenças de comportamentos sexuais entre eles.

### Contribuições para área

A contribuição deste estudo favorece a identificação das populações mais vulneráveis à infecção pelo HIV nos Centros de Testagem e Aconselhamento, considerando que esses serviços representam portas de entrada para a população, viabilizando tanto o atendimento coletivo quanto individual. Esse aspecto é fundamental para o diagnóstico oportuno, o início precoce do tratamento e o acompanhamento da carga viral, com vistas à sua supressão. Além disso, o estudo possibilita a construção do conhecimento sobre os modos de transmissão do HIV, contribuindo para a adoção de estratégias eficazes na redução da sua disseminação.

## CONCLUSÕES

A partir do reconhecimento dos fatores associados ao HIV entre jovens, estratégias direcionadas à prevenção, diagnóstico e tratamento oportuno da infecção devem ser priorizadas principalmente junto àqueles que são homens, profissionais do sexo e que realizam práticas sexuais desprotegidas e não vaginais. Tais estratégias devem estar alinhadas à Prevenção Combinada ao HIV e à Linha do Cuidado contínuo às pessoas que vivem com HIV.

## Data Availability

Os dados de pesquisa estão disponíveis no corpo do artigo.
